# Postoperative acute kidney injury after on-pump cardiac surgery in patients with connective tissue disease

**DOI:** 10.3389/fcvm.2023.1266549

**Published:** 2023-11-01

**Authors:** Xue Zhang, Qi Miao, Chunhua Yu, Yuelun Zhang, Di Wu, Yajie Tian, Hanchen Li, Chunrong Wang

**Affiliations:** ^1^ Department of Anesthesiology, Peking Union Medical College Hospital, Chinese Academy of Medical Sciences, Beijing, China; ^2^ Department of Cardiac Surgery, Peking Union Medical College Hospital, Chinese Academy of Medical Sciences, Beijing, China; ^3^ Medical Research Center, Peking Union Medical College Hospital, Chinese Academy of Medical Sciences, Beijing, China; ^4^ Department of Rheumatology, Peking Union Medical College Hospital, Chinese Academy of Medical Sciences, Beijing, China

**Keywords:** acute kidney injury, cardiac surgery, connective tissue disease, systemic lupus erythematosus, body mass index

## Abstract

**Objective:**

Patients with connective tissue disease have a poor prognosis after receiving cardiac surgery. This study described the clinical scenarios and investigated factors correlated with acute kidney injury (AKI) after on-pump cardiac surgery in patients with systemic lupus erythematosus (SLE) or vasculitis.

**Methods:**

Patients with SLE or vasculitis who underwent on-pump cardiac surgery from March 2002 to March 2022 were enrolled, while patients with preoperative renal dysfunction were excluded. AKI was defined according to the Kidney Disease: Improving Global Outcomes (KDIGO) criteria. Uni- and multivariable analyses were performed to identify potential factors associated with postoperative AKI.

**Results:**

Among 123 patients enrolled, 39 (31.7%) developed AKI within seven days after receiving on-pump cardiac surgery. Four patients died in the hospital, resulting in an overall in-hospital mortality of 3.3%, and all deaths occurred in the AKI group. Patients in the AKI group also had longer ICU stays (median difference 3.0 day, 95% CI: 1.0–4.0, *P* < 0.001) and extubation time (median difference 1.0 days, 95% CI: 0–2.0, *P* < 0.001) than those in the non-AKI group. Multivariable logistic regression revealed that BMI over 24 kg/m^2^ (OR: 3.00, 95% CI: 1.24–7.28) and comorbid SLE (OR: 4.73, 95% CI: 1.73–12.93) were independently correlated with postoperative AKI.

**Conclusion:**

Factors potentially correlated with AKI following on-pump cardiac surgery in patients with connective tissue disease were explored. Clinicians should pay more attention to preoperative evaluation and intraoperative management in patients with risk factors.

## 1. Introduction

Patients undergoing on-pump cardiac surgery are susceptible to postoperative acute kidney injury (AKI), with the reported incidence varying from 5% to 42.3% (1, 2). AKI following cardiac surgery is known to be independently associated with an increased risk of chronic kidney disease, an extended recovery time and increased health care costs and is believed to be the strongest predictor of mortality following surgery (3, 4).

Cardiac involvement, such as ascending aortic involvement, cardiac valvular involvement, and coronary artery involvement, is fairly frequent in patients with connective tissue disease (CTD) (5–8). These patients can have persistent clinical symptoms; therefore, surgical interventions are always indicated (9). Numerous studies have focused on analyzing risk factors for cardiac surgery–associated AKI; however, to our knowledge, there have been no studies exploring the preoperative predictors of cardiac surgery–associated AKI in patients with CTD due to the rarity of these diseases.

This single-center observational study aimed to describe the prevalence and prognosis of cardiac surgery–associated AKI and to investigate the potential predictors of cardiac surgery–associated AKI in patients with systemic lupus erythematosus (SLE) or vasculitis undergoing on-pump cardiac surgery.

## 2. Materials and methods

### 2.1. Patient enrollment

This study was retrospectively conducted at a tertiary teaching hospital in Beijing, China. Ethics approval was obtained from the Peking Union Medical College Hospital Institutional Review Board (S-K2063, 15 March 2022). No informed consent was required because the study was retrospective and the data were anonymized. The hospital medical record system was searched, and adult patients diagnosed with vasculitis (mainly including Takayasu arteritis and Behçet's disease) or SLE who underwent cardiac surgery from March 2002 to March 2022 were consecutively enrolled. All patients were diagnosed by an experienced rheumatologist. The following exclusion criteria were applied: (1) diagnosis of chronic kidney dysfunction at the time of surgery [estimated glomerular filtration rate (eGFR) <60 ml/min/1.73 m^2^]; (2) need for renal replacement therapy prior to cardiac surgery; and (3) intraoperative death.

### 2.2. Data collection

All members of the research group signed confidentiality agreements before receiving the data. To ensure validity and reliability throughout the study, all the members received uniform training on data extraction. Data were collected from the medical recording system.

Demographic data, including sex, age, height, weight, body mass index (BMI), smoking and drinking history, American Society of Anesthesiologists physical status and New York Heart Association heart functional class, were collected. Preoperative data were extracted, including comorbidities [hypertension (defined as systolic blood pressure over 130 mmHg or diastolic blood pressure over 80 mmHg), diabetes mellitus (defined as glycated hemoglobin over 6.5%, or fasting plasma glucose over 7 mmol/L, or 2-hour plasma glucose over 11.1 mmol/L during an oral glucose tolerance test, or random plasma glucose over 11.1 mmol/L in patient with classic symptoms of hyperglycemia or hyperglycemic crisis), arrhythmia, cerebrovascular disease], and course of CTD. Furthermore, preoperative laboratory test results [complete blood count, liver and renal function, coagulation test, erythrocyte sedimentation rate (ESR), and C-reactive protein (CRP)] were also extracted. The intraoperative data we collected included the type of surgery, time of surgery, time of cardiopulmonary bypass, time of aortic cross-clamping, quantities of blood component infusion, and vasoactive drug dose. The vasoactive-inotropic score was calculated at operating theater discharge based on a weighted sum of all administered inotropes and vasoconstrictors, reflecting pharmacological support of the cardiovascular system (10). Prognostic information was also collected, including extubation time, intensive care unit length of stay, death, extracorporeal membrane oxygenation support, paravalvular leak, reoperation, cerebrovascular accident, and infection.

AKI was defined according to the Kidney Disease: Improving Global Outcomes (KDIGO) criteria, as follows: an increase in serum creatinine concentration ≥0.3 mg/dl (26.5 µmol/L) within 48 h, or an increase in serum creatinine concentration ≥1.5 times of baseline that is known or presumed to have occurred within the prior 7 days, or urine output <0.5 ml/kg/h for 6 h (11, 12). The KDIGO criteria for AKI staging demonstrate greater sensitivity in detecting AKI and predicting associated in-hospital mortality than either the risk of renal dysfunction, injury to the kidney, failure of kidney function, loss of kidney function, and end-stage kidney disease criteria or the Acute Kidney Injury Network criteria (13). The eGFR value was calculated was estimated according to the Chronic Kidney Disease Epidemiology Collaboration equation (14).

### 2.3. Statistical analysis

The demographic and clinical characteristics of the patients were described as follows. The normality of the continuous variables were assessed using the Shapiro–Wilk test. Continuous variables were expressed as the mean ± standard deviation or medians (25th–75th percentiles). Categorical variables are expressed as case numbers and percentages. Normally distributed continuous variables were analyzed using an independent *t*-test, whereas nonnormally distributed continuous variables using the Mann‒Whitney *U*-test. Categorical variables were compared using the chi-square (*χ*^2^) test by default or Fisher's exact test if the expected count in each cell of the table was less than 5. Continuous variables were transformed into categorical variables based on standard cutoff values used in clinical practice or according to the clinicians' experience (including BMI, hemoglobin, cardiac troponin I (cTnI), N-terminal pro-B-type natriuretic peptide (NT-proBNP), ESR, and CRP). Median differences and corresponding 95% confidence intervals were calculated using Hodges-Lehmann method on the basis of Mann–Whitney *U*-test.

Clinical factors potentially correlating with postoperative AKI occurrence were all first analyzed using univariable analysis. Variables with potential associations (*P* < 0.1) in the univariable analysis were incorporated into the ultimate multivariable model. A logistic regression model with the enter method was then built to screen the correlated factors, in which the occurrence of postoperative AKI was analyzed as the binary outcome.

Another 2 sensitive analyses were additional performed to examine the robustness of the results: (1) Besides patients with eGFR of 60 ml/min/1.73 m^2^ and greater, those with mild to moderate kidney dysfunction (eGFR of 45–60 ml/min/1.73 m^2^) were also incorporated into the analysis; (2) the multivariable analysis was further conducted among those with both NT-proBNP and cTnI available prior to surgery.

A two-sided *P*-value less than 0.05 was regarded as statistically significant. SPSS (IBM SPSS statistics Version 26, Chicago, IL, USA) was used for the statistical analysis of the data.

## 3. Results

### 3.1. Patient demographic characteristics and incidence of AKI occurrence

A total of 152 adult patients with SLE or vasculitis received on-pump cardiac surgery from March 2002 to March 2022 in our hospital. Twenty-seven of them had preoperative chronic kidney dysfunction or were supported by renal replacement therapy, and two patient died intraoperatively, such that a total of 29 patients were excluded. Ultimately, there were 123 patients enrolled in our study. Ninety-two (74.8%) patients had accompanying vasculitis, including 55 (44.7%) patients with Takayasu arteritis, 31 (25.2%) patients with Behçet's disease and 6 (4.8%) patients with other types of vasculitis. The other 31 (25.2%) patients had accompanying SLE. The AKI incidence in males and females was 32.0% and 31.5%, respectively (*P* = 0.954).

Based on the KDIGO definition, AKI occurred in 39 (31.7%) patients within seven days postoperatively, and these patients were defined as the AKI group. The other 84 (68.3%) patients did not develop AKI and were defined as the non-AKI group. The demographic data and clinical characteristics of the patients are presented in Table 1. Patients in the AKI group were older than patients in the non-AKI group [44 years (32.0–53.0) vs. 40.0 years (29.0–49.0)], though no significant difference was revealed (median difference 3.0 years, 95% CI: 1.0–8.0, *P* = 0.158). Obesity (BMI ≥ 24 kg/m^2^) was more prevalent in the AKI group than in the non-AKI group (OR: 2.0, 95% CI: 0.9–4.5, *P* = 0.074), and comorbid SLE as opposed to vasculitis was significantly more common in the AKI group than in the non-AKI group (OR: 2.7, 95% CI: 1.1–6.2, *P* = 0.021).

**Table 1:** Preoperative baseline characteristics of enrolled patients with and without postoperative acute kidney injury.

Characteristics	All patients *N* = 123	Non-AKI group *N* = 84	AKI group *N* = 39	OR/mean difference/median difference (95% CI)	*P*-value
Sex					
Male	50 (37.6%)	34 (40.5%)	16 (41.0%)	1.0 (0.5–2.2)	0.954
Female	73 (62.4%)	50 (59.5%)	23 (59.0%)		
Age (yr)	41.0 (30.0–50.0)	40.0 (29.0–49.0)	44 (32.0–53.0)	3.0 (−1.0–8.0)	0.158
Height (cm)	165.4 ± 6.6	165.4 ± 6.4	165.5 ± 7.2	0.2 (−2.4–2.7)	0.893
Weight (kg)	60.8 ± 11.2	60.3 ± 10.6	61.8 ± 12.4	1.5 (−3.1–6.1)	0.482
BMI (kg/m^2^)	21.6 (19.8–24.6)	21.4 (19.9–24.2)	22.5 (19.1–26.0)	0.9 (−0.6–2.4)	0.212
BMI ≥ 24 kg/m^2^	40 (32.5%)	23 (27.4%)	17 (43.6%)	2.0 (0.9–4.5)	0.074
Smoking history	24 (19.5%)	18 (21.4%)	6 (15.4%)	0.7 (0.2–1.8)	0.431
Drinking history	15 (12.2%)	12 (14.3%)	3 (7.7%)	0.5 (0.1–1.9)	0.298
Comorbidities					
Hypertension	28 (22.8%)	19 (22.6%)	9 (23.1%)	1.0 (0.4–2.5)	0.955
Diabetes mellitus	6 (4.9%)	4 (4.8%)	2 (5.1%)	1.1 (0.2–6.2)	0.930
Arrhythmia	3 (2.4%)	2 (2.4%)	1 (2.6%)	1.1 (0.1–12.3)	0.951
Cerebrovascular disease	10 (8.1%)	7 (8.3%)	3 (7.7%)	0.9 (0.2–3.8)	0.904
Type of CTD					
SLE	31 (25.2%)	16 (19.0%)	15 (38.5%)	2.7 (1.1–6.2)	0.021
Vasculitis	92 (74.8%)	68 (81.0%)	24 (61.5%)		
Course of CTD (months)	24 (8–108)	24 (6–117)	24 (24–96)	5.0 (−1.0–19.0)	0.241
Type of surgery					
Valvuloplasty/Valve replacement	56 (45.5%)	39 (46.4%)	17 (43.6%)	–	0.640
Aortic root/Ascending aorta replacement	17 (13.8%)	9 (10.7%)	8 (20.5%)		
CABG	19 (15.4%)	14 (16.7%)	5 (12.8%)		
Combined surgery	22 (17.9%)	15 (17.9%)	7 (17.9%)		
Others	9 (7.3%)	7 (8.3%)	2 (5.1%)		
ASA PS					
I-II	10 (8.1%)	6 (7.1%)	4 (10.3%)	0.7 (0.2–2.5)	0.557
III-IV	113 (91.9%)	78 (92.9%)	35 (89.7%)		
NYHA grade					
I-II	58 (48.2%)	43 (52.2%)	15 (39.5%)	1.7 (0.8–3.6)	0.188
III-IV	65 (52.8%)	41 (48.8%)	24 (61.5%)		

AKI, acute kidney injury; OR, odds ratio; CI, confidence interval; BMI, body mass index; CTD, connective tissue disease; SLE, systemic lupus erythematosus; CABG, coronary artery bypass grafting; ASA PS, American society of anesthesiologists physical status; NYHA, New York heart association.

To evaluate the proportion of patients with active/non-active inflammation state, we checked patients' preoperative consultation performed by department of rheumatology. Patients without preoperative consultation were evaluated retrospectively by rheumatologist. CTD activity was checked by SLEDAI (SLE disease activity index), BDCAF (Behçet's disease current activity form), and Kerr index for patients with SLE, Behçet's disease, and Takayasu arteritis, respectively. 116 patients had preoperative disease activity result. 39.5% (32/81) patients in the AKI group and 45.7% (16/35) patients in the non-AKI group were in active state, and the difference was not statistically significant (*P* = 0.545).

Among 123 patients enrolled, 87 (70.7%) patients were under medication treatment (37 patients used glucocorticoid, 7 patients used immunosuppressant, and 43 patients were administered both glucocorticoid and immunosuppressant). 32 (26.0%) patients did not receive medical anti-inflammation treatment, and 4 patients' treatment information were not available.

### 3.2. Preoperative laboratory tests and intraoperative data

At baseline, no significant difference was revealed regarding serum creatinine level [69.2 (18.1) µmol/L vs. 68.8 (15.2) µmol/L, *P* = 0.891] or eGFR level [101.1 (21.3) ml/min/1.73 m^2^ vs. 104.6 (17.6) ml/min/1.73 m^2^, *P* = 0.343] between the AKI group and the non-AKI group. In comparison with the non-AKI group (*N* = 64), the NT-proBNP level was significantly higher in the AKI group (*N* = 27) [574.0 ng/L (180.0–1,722.3) vs. 1,720.0 ng/L (830.0–5,223.0); median difference 1,012.0 ng/L, 95% CI: 403.0–2,594.0, *P* = 0.002]. The univariable analyses of intraoperative data did not show any significant differences between the two groups (all *P* levels >0.05). The results are listed in detail in Tables 2, 3.

**Table 2:** Comparison of preoperative laboratory test results between patients with and without postoperative AKI.

Laboratory test	All patients *N* = 123	Non-AKI group *N* = 84	AKI group *N* = 39	OR/mean difference/median difference (95% CI)	*P*-value
Anemia (<120 g/L in males, <110 g/L in females)	36 (29.3%)	21 (25.0%)	15 (38.5%)	1.9 (0.8–4.2)	0.127
White blood cell (×10^9^/L)	7.3 (5.7–9.7)	7.5 (5.7–10.2)	7.2 (5.7–9.3)	−0.2 (−1.3–0.9)	0.744
Percentage of neutrophils (%)	67.7 (12.0)	67.3 (11.8)	68.9 (12.4)	2.7 (−2.6–7.0)	0.463
Platelet (×10^9^/L)	205.0 (149.0–266.0)	201.5 (151.8–267.5)	205.0 (144.0–251.0)	2.0 (−30.0–34.0)	0.896
Alanine aminotransferase (U/L)	18.0 (12.0–28.0)	17.0 (12.0–26.8)	19.0 (14.0–36.0)	2.0 (−2.0–6.0)	0.308
Albumin (g/L)	38.0 (35.0–40.0)	39.0 (36.0–40.0)	36 (33–41)	−1.0 (−3.0–1.0)	0.165
Serum creatinine (µmol/L)	68.9 (16.1)	68.8 (15.2)	69.2 (18.1)	0.4 (−5.8–6.6)	0.891
eGFR (ml/min/1.73 m^2^)	103.5 (18.9)	104.6 (17.6)	101.1 (21.3)	−3.5 (−10.7–3.8)	0.343
cTn I (ng/ml)	0.02 (0.02–0.05) (*N* = 94)	0.02 (0.02–0.03) (*N* = 64)	0.02 (0.02–0.13) (*N* = 30)	0.002 (0–0.017)	0.076
Increased cTn I (>0.056 ng/ml)	19 (20.2%)	10 (15.6%)	9 (30.0%)	2.3 (0.8–6.5)	0.106
NT-proBNP (ng/L)	912.0 (228.0–2,889.0) (*N* = 91)	574.0 (180.0–1,722.3) (*N* = 64)	1,720.0 (830.0–5,223.0) (*N* = 27)	1,012.0 (403.0–2,594.0)	0.002
Increased NT-proBNP (>125 ng/L)	77 (84.6%)	52 (81.3%)	25 (92.6%)	2.9 (0.6–13.9)	0.171
Prothrombin time (s)	11.9 (11.2–12.8)	11.9 (11.2–12.7)	12.1 (11.4–13.1)	0.3 (−0.2–0.7)	0.206
Activated partial thromboplastin time (s)	27.9 (25.2–31.6)	27.7 (25.2–31.6)	28.4 (25.1–32.5)	0.2 (−1.5–2.0)	0.826
Fibrinogen (mg/dl)	2.7 (2.2–3.6)	2.7 (2.1–3.8)	2.8 (2.2–3.6)	0.2 (−0.2–0.6)	0.382
ESR (mm)	13.0 (7.0–28.0)	13.0 (7.0–27.8)	13.0 (8.0–36.0)	2.0 (−2.0–6.0)	0.377
Elevated ESR (>15 mm)	53 (43.1%)	35 (41.7%)	18 (46.2%)	0.8 (0.4–1.8)	0.640
CRP (mg/dl)	3.5 (0.9–9.3)	3.5 (1.0–9.2)	3.5 (0.6–10.6)	0.0 (−1.1–2.3)	0.862
Elevated C-reactive protein (>8 mg/dl)	36 (29.3%)	24 (28.6%)	12 (30.8%)	0.9 (0.4–2.1)	0.803

AKI: acute kidney injury; OR: odds ratio; CI: confidence interval; eGFR: estimated glomerular filtration rate; cTnI, cardiac troponin I; NT-proBNP: N-terminal pro-B-type natriuretic peptide; ESR, elevated erythrocyte sedimentation; CRP, elevated C-reactive protein.

**Table 3:** Comparison of intraoperative data between patients with and without postoperative acute kidney injury.

Intraoperative data	All patients *N* = 123	Non-AKI group *N* = 84	AKI group *N* = 39	OR/Median difference (95% CI)	*P*-value
CPB time (min)	128.5 (95.0–167.0)	128.5 (99.3–173.3)	128.5 (88.0–166.0)	8.0 (−10.5–27.0)	0.389
ACx time (min)	83.0 (58.0–106.0)	83.0 (58.3–109.0)	83.0 (58.0–100.0)	2.0 (−10.0–17.0)	0.659
Surgery time (min)	310.0 (260.0–420.0)	317.5 (260.0–420.0)	300.0 (265.0–410.0)	10.0 (−25.0–50.0)	0.621
Intraoperative RBC transfusion	58 (47.2%)	44 (52.4%)	14 (35.9%)	0.5 (0.2–1.1)	0.088
RBC transfusion >4 u	26 (21.1%)	19 (22.6%)	7 (17.9%)	1.4 (0.5–3.5)	0.555
Plasma transfusion	86 (69.9%)	63 (75.0%)	23 (59.0%)	0.5 (0.2–1.1)	0.071
Plasma transfusion >400 ml	24 (19.5%)	18 (21.4%)	6 (15.4%)	0.7 (0.2–1.8)	0.431
Platelet transfusion	60 (48.8%)	42 (50.0%)	18 (46.2%)	1.2 (0.5–2.5)	0.691
Platelet transfusion (ml)	0 (0–200.0)	100.0 (0–200.0)	0 (0–200.0)	0.0 (0.0–0.0)	0.741
Vasoactive-inotropic score*	10 (6–20)	10 (7–20)	20 (6–20)	0.0 (−3.0–4.0)	0.729
Vasoactive-inotropic score >10	74 (60.2%)	50 (59.5%)	24 (61.5%)	0.9 (0.4–2.0)	0.832

AKI, acute kidney injury; OR, odds ratio; CI, confidence interval; CPB, cardiopulmonary bypass; ACx, aortic cross-clamping; RBC, red blood cell.

* Vasoactive-inotropic score was calculated as: dopamine (µg/kg/min) + dobutamine (µg/kg/min) + [100 × epinephrine (µg/kg/min)] + [100 × norepinephrine (µg/kg/min)] + [10,000 × vasopressin (U/kg/min)].

### 3.3. Factors correlated with postoperative AKI

A multivariable logistic regression test was performed to identify potential correlated factors for AKI based on the comparison between the AKI and non-AKI groups. The analysis results (Table 4) showed that BMI over 24 kg/m^2^ (OR: 3.00, 95% CI: 1.24–7.28) and comorbid SLE (OR: 4.73, 95% CI: 1.73–12.93) were independently correlated with postoperative AKI occurrence.

**Table 4:** Risk factors investigated on multivariable models considering confounders with a *P*-value of <0.1 on individual univariate analysis.

Potential correlated factors	Adjusted OR (95% CI)	Adjusted-*P*-value
Platelet count (per 10^9^/L increment)	1.00 (1.00–1.01)	0.108
BMI ≥ 24 kg/m^2^ (yes vs. no)	3.00 (1.24–7.28)	0.015
SLE (yes vs. no)	4.73 (1.73–12.93)	0.002

With adjustment for sex, age, BMI ≥ 24 kg/m^2^, baseline eGFR, smoking history, drinking history, comorbidities (hypertension, diabetes mellitus, arrythmia and cerebrovascular disease and anemia), types of connective tissue disease (SLE vs. vasculitis), ASA PS (I-II vs. III-IV), NYHA (I-II vs. III-IV), laboratory biomarkers (white blood cell count, percentage of neutrophils, platelet count, alanine aminotransferase and albumin), coagulation tests (prothrombin time, activated partial thromboplastin time and fibrinogen levels), ESR > 15 mm, CRP > 8 mg/dl, surgical information (aortic cross-clamping time and surgical types), intra-operative transfusion (red blood cells transfusion ≥4 u, fresh frozen plasma transfusion ≥400 ml and platelet transfusion) and vasoactive-inotropic score ≥10.

OR, odds ratio; CI, confidence interval; BMI, body mass index; eGFR, estimated glomerular filtration rate; SLE, systemic lupus erythematosus; ASA PS, American society of anesthesiologists physical status; NYHA, New York heart association; ESR, elevated erythrocyte sedimentation; CRP, C-reactive protein.

The sensitivity analysis performed in patients with eGFR of 45 ml/min/1.73 m^2^ and greater indicated that, in the context of AKI incidence of 34.1% (45/132), risk factors associated with postoperative AKI kept unaltered (BMI over 24 kg/m^2^ OR: 2.59, 95% CI: 1.14–5.84, *P* = 0.022; SLE OR: 4.97, 95% CI: 1.94–12.7, *P* = 0.001). Another sensitivity analysis restricted in those with both baseline NT-proBNP and cTnI available (*N* = 88) also showed the same results.

### 3.4. In-hospital outcome and prognosis

Four patients died in the hospital, resulting in an overall in-hospital mortality of 3.3%. All deaths occurred in the AKI group, and the mortality difference between the two groups was significant (OR: 1.1, 95% CI: 1.0–1.2, *P* = 0.003). Patients in the AKI group had a longer length of stay in intensive care unit (median difference 3.0 day, 95% CI: −1.0–4.0, *P* < 0.001) and time to extubation (median difference 1.0 days, 95% CI: 0–2.0, *P* < 0.001) than those in the non-AKI group. Regarding postoperative complications, more cerebrovascular accidents occurred in the AKI group than in the non-AKI group. The results are shown in Table 5.

**Table 5:** Postoperative outcomes between patients with and without AKI.

	All patients (*N* = 123)	Non-AKI group (*N* = 84)	AKI group (*N* = 39)	Median difference/OR (95% CI)	*P*-value
Time to extubation (days)	2.0 (1.0–3.0)	1.0 (1.0–2.0)	2.0 (1.0–8.0)	1.0 (0–2.0)	<0.001
Length of stay in intensive care unit (days)	3.0 (0.0–5.0)	2.0 (0.0–3.0)	5.0 (3.0–7.0)	3.0 (1.0–4.0)	<0.001
Postoperative complications					
Death	4 (3.3)	0	4 (10.3%)	1.1 (1.0–1.2)	0.003
ECMO support	2 (1.6)	0	2 (5.1%)	1.1 (1.0–1.1)	0.099
Paravalvular leak	2 (1.6)	1 (1.2)	1 (2.6)	2.2 (0.1–35.9)	0.535
Reoperation	7 (5.7)	4 (4.8)	3 (7.7)	1.7 (0.4–7.8)	0.678
Cerebrovascular accident	3 (2.4)	0 (0)	3 (7.7)	1.1 (1.0–1.2)	0.03
Infection	16 (13.0%)	9 (10.7%)	7 (17.9%)	1.8 (0.6–5.3)	0.267

AKI, acute kidney injury; OR: odds ratio; ECMO: extracorporeal membrane oxygenation.

## 4. Discussion

Perioperative management of patients combined with CTD was special in many aspects. Oral glucocorticoids are the most commonly used drugs for treatment of combined CTD. Chronic glucocorticoid therapy can suppress the hypothalamic-pituitary-adrenal axis, and result in unappropriated response during surgery stress. Therefore, perioperative intravenous glucocorticoid replacement was very important in these patients, which should be directed by experienced physician. SLE was always associated with multiple organ dysfunction due to systemic involvement, so that perioperative evaluation and monitoring of organ function was necessary. Vascular wall and valvular tissue in patients with vasculitis were always fragile, raising difficulty in surgical suture. Therefore, the risk of postoperative bleeding as well as perivalvular leak was higher in these patients. Anesthesia doctors should pay more attention on coagulation function correction. Intraoperative transesophageal echocardiography could help evaluate valvular structure and function after cardiopulmonary bypass and was highly recommended in patients with CTD.

The kidney is frequently involved in autoimmune CTD. Many known mechanisms of renal involvement have been discovered, such as antibody-mediated glomerular injury, small-vessel vasculitis, and pharmacotherapy-related kidney injury, have been discovered (15–17); through mechanisms such as these, patients with CTD are more vulnerable to postoperative renal injury than those without CTD. Alliu and colleagues found that CTD patients undergoing percutaneous coronary intervention had a higher incidence of postoperative AKI and a longer hospital stay than patients without CTD (18).

Our study is the first to focus on patients with CTD undergoing on-pump cardiac surgery, and the incidence of AKI in our cohort was 31.7%. It should be noted that patients with preoperative eGFR < 60 ml/min/2.73 m^2^ or a need for renal replacement therapy were excluded in our primary anslysis so that preexisting moderate and severe renal dysfunction would not influence the results; these criteria were not applied in other similar studies. Therefore, the incidence of postoperative AKI in patients with CTD might have been even higher if these patients were enrolled. Our study also demonstrated that higher mortality, later extubation, and longer ICU LOS were all significantly associated with postoperative AKI occurrence; therefore, postoperative AKI in CTD patients undergoing cardiac surgery should be scrutinized.

Risk factors for cardiac surgery–associated AKI have already been well studied (13, 19–21). Wang's team from West China Hospital even established a predictive model incorporating 10 predictors; this model could be used not only for predicting AKI after on-pump cardiac surgery but also for predicting the severity of AKI, and external validation was successful (1). Wang and colleagues from Peking Union Medical College Hospital established a model for predicting AKI after pericardiectomy (22). However, due to the rarity of comorbid CTD, risk factors for postoperative AKI occurrence in patients with CTD undergoing on-pump cardiac surgery were not available in the literature.

We found some potential independent risk factors in our study. First, among patients with CTD, we found that overweight patients with BMI ≥ 24 kg/m^2^ had nearly a threefold increase in postoperative AKI risk. In fact, several studies have reported a relationship between obesity and AKI, although the definition of obesity was inconsistent (23, 24). Shi et al. demonstrated the association between overweight and cardiac surgery–associated AKI occurrence. They not only found a significantly increased risk of developing cardiac surgery–associated AKI in patients with BMI > 24 kg/m^2^ but also confirmed by meta-analysis that overweight patients had a higher AKI risk than normal-weight patients (25). Cardiac patients with excessive BMI are at an increased risk of AKI, owing to their disproportionately higher burden of comorbidities such as hypertension, diabetes mellites and hyperlipidemia. Underlying structural changes that occur in the kidneys may also contribute to postoperative AKI in obese patients (26). What's more, obesity may significantly alter renal hemodynamics. Increased renal plasma flow and glomerular filtration rate may lead to high filtration syndrome, making the kidney prone to damage (27).

Interestingly, Schvartz conducted research in ST-segment elevation myocardial infarction patients receiving percutaneous catheterization intervention and found that BMI had a significant protective effect against mortality among patients with AKI (28). Marzo also demonstrated that higher BMI was associated with better survival in transcatheter aortic valve replacement patients who developed AKI (29). This phenomenon has been observed in many clinical scenarios and is called “the obesity paradox”: obese patients have superior outcomes even though obesity is associated with an increased incidence of AKI (30, 31). Further studies should investigate the relationship between obesity and mortality in CTD patients who develop AKI after undergoing cardiac surgery.

Second, although our study excluded patients with preoperative renal dysfunction to avoid any influence of preexisting kidney injury, our results still illustrated that patients with SLE were more likely to have postoperative AKI than patients with vasculitis. SLE is a chronic autoimmune disease of unknown cause that can affect virtually any organ of the body; therefore, the kidneys of SLE patients might face an increased risk of lasting damage as a result of surgery. Clinically apparent kidney involvement is reported to occur in approximately 50% of SLE patients and is a significant cause of morbidity and mortality, but its clinical manifestations can be highly variable (32). A more comprehensive preoperative renal function evaluation including tests such as urinalysis, quantitation of proteinuria, and estimation of the glomerular filtration rate should be considered. Intraoperative management such as volume monitoring and perfusion pressure control should also be further refined and individualized in patients with SLE.

The rate of AKI appeared in a decreased manner from aortic root/ascending aorta replacement procedures (47.1%) to combined surgery (31.8%) or valvuloplasty/valve replacement procedures (30.4%) to coronary artery bypass graft (26.3%). The effective improvement of renal perfusion may be secondary to swift and rapid alleviation of myocardial ischemia following coronary revascularization. Furthermore, there exists no surprise that renal impairment after aortic involving procedures was the most obtrusive, which was also highlighted by a previous study conducted within a large-cohort of patients (33). In ours postsurgical AKI was not associated with sex disparity, though several publications, in sharp contrast, ever ascertained that female could be either a risk factor (34, 35) or a protective factor (36, 37) towards any-stage or severe AKI. It should be pointed out that incoherent conclusion regarding the linkage of sex and AKI may be, to some extent, a reflection of sex-related comobidities burden disparity and AKI definitions variance.

Several limitations should be considered when interpreting the present results. First, this was a single-center retrospective study; therefore, it has limitations in terms of the applicability of its results to other clinical settings. Second, we only included patients with SLE or vasculitis in our study. Patients with other types of CTD might have completely different clinical features, and their risk factors require further investigation. Third, patients with preoperative renal dysfunction were excluded from our study; thus, the results could only partially reflect the overall scenario. Last but not least, the rarity of CTD led to a very small sample size, which gave the study limited statistical power to detect potential correlated factors. This also resulted in a compromised ability to appropriately control the confounding effects using multivariable regression. Confounding effects cannot be fully excluded in this study; hence, the results should be interpreted with caution. Multicenter studies are required to further validate these findings.

In conclusion, CTD patients undergoing on-pump cardiac surgery were susceptible to postoperative AKI, which was associated with increased mortality and a worsened prognosis. We found that patients with BMI over 24 kg/m^2^ or comorbid SLE were especially vulnerable to postoperative AKI after receiving on-pump cardiac surgery. Clinicians should pay more attention to preoperative evaluation and intraoperative management in patients with risk factors for postoperative AKI.

## Data Availability

The raw data supporting the conclusions of this article will be made available by the authors, without undue reservation.
